# Psychometrics of the Spanish Version of the Screen for Adult Anxiety Related Disorders (SCAARED)

**DOI:** 10.3389/fpsyt.2021.589422

**Published:** 2021-02-11

**Authors:** Sarah Sánchez-Cueva, Yurena Alonso-Esteban, Patricio Sánchez-Cueva, Boris Birmaher, Francisco Alcantud-Marín

**Affiliations:** ^1^Department of Developmental and Educational Psychology, University of Valencia, Valencia, Spain; ^2^College of Medicine, Pennsylvania State University, Hershey, PA, United States; ^3^Department of Psychiatry, University of Pittsburgh Medical Center, Pittsburgh, PA, United States

**Keywords:** anxiety disorders, anxiety measures, rating scales, tools translation, measure of anxiety in adults

## Abstract

**Objectives:** To translate and validate the Screen for Adult Anxiety Related Disorders (SCAARED) questionnaire into Spanish.

**Method:** The original SCAARED was translated into Spanish and administered to a non-clinical sample of 131 university students (92.4% women, mean age 22 years) in Valencia, Spain. To assess the concurrent validity of the SCAARED, the Depression, Anxiety and Stress Scale−21(DASS) and the Beck's Anxiety Inventory (BAI) were also administered. Test-retest reliability was evaluated 2 weeks after the first administration.

**Results:** The internal consistency of SCAARED was high (α = 0.91) and the stability of the measurement was also high (test-retest 0.81). The results of the Exploratory Factor Analysis showed four factors comparable to the original SCAARED (generalized anxiety disorder, social phobia disorder, panic disorder, and separation anxiety disorder). The Area Under the Curve was excellent (0.88).

**Conclusions:** The Spanish version of the SCAARED showed good psychometric properties comparable to the original SCAARED suggesting that it may be a useful instrument to screen for anxiety disorders in Spanish-speaking adult populations. Future studies are needed to replicate these findings in larger community and clinical samples.

## Introduction

Anxiety disorders including generalized anxiety disorder, social anxiety disorder, panic disorder, agoraphobia and specific phobia, are among the most common psychiatric disorders in youths and adults with 4–25% of people suffering from one or more anxiety disorders in their lives (1, 2).

There has been a growing interest in research on anxiety disorders in the last decade, partly due to greater recognition of their burden and the impact of untreated illnesses (3). Results from a recent review and meta-analysis indicate that the majority of anxiety disorders tend to have an early onset, generally in childhood or early adolescence (4, 5), and endure over time if not properly treated. Anxiety disorders experienced before or during early adulthood have been associated with poor psychosocial functioning (e.g., work), poor health, low life satisfaction, and less social relationships during adulthood (6, 7). In addition, there is substantial evidence to suggest that individuals with anxiety disorders are at risk to develop substance abuse (5, 8); chronical medical illness (8); depressive disorders (9, 10); suicide-related behaviors or other risky behaviors (11).

Unfortunately, anxiety disorders may be unrecognized, particularly when is comorbid with other disorders such major depression, making treatment ineffective. The high prevalence of anxiety disorders among youth and adults and the resulting consequences recommend early detection to identify anxiety symptoms in these age groups. One of the factors that influence the under recognition of anxiety disorders is the limitations of current screening instruments (12, 13) in typically developing adult's populations (14) and among people with neurodevelopmental disorders (15). The use of structured (or semi-structured) interviews to evaluate anxiety disorders is the procedure of choice for establishing the diagnosis of an anxiety disorder (16), but is time-consuming and requires extensive training from either Primary Health Care professionals, clinical psychologists, and researchers. Consequently, self-report measures are the most common method of anxiety assessment (12, 17). Still, access to good screening instruments (with good levels of reliability, validity, and diagnostic discrimination), including formats for various informants (e.g., parents, teachers, self-reporters) and are affordable and adapted to Spanish-speaking populations, is often limited.

Currently, the most popular instruments for assessing anxiety in adults, include the State-Trait Anxiety Inventory (STAI) (18) and the Beck Anxiety Inventory (BAI) (19), two empirically and widely validated instruments used in psychological research and clinical practice in Spain (20, 21). Also, the Depression, Anxiety, and Stress Scale (DASS-21) (22), has shown promise in the screening for anxiety symptoms. Several other validated and reliable anxiety measures for specific anxiety disorders exist including, among others, the Generalized Anxiety Disorder Questionnaire and Generalized Anxiety Disorder-7 Scale (GAD-Q-IV & GAD-7) (23); the Social Phobia Inventory (SPIN) (24), the Liebowitz Social Anxiety Scale (LSAS) (25); and the Panic Disorder Severity Scale (PDSS) (26).

One of the limitations of the above scales is that they mainly assess one or two specific anxiety disorders. Although informative, this may be problematic because anxiety disorders usually are comorbid within themselves (27). Recently, a screen for all DSM-5 (28) was developed, the Screen for Adult Anxiety Related Emotional Disorders (SCAARED) (29). The SCAARED is a 44-item self-report instrument that was adapted from the youth instrument, the Screen for Children Anxiety Related Emotional Disorders (SCARED) (30, 31), a rating scale developed to screen for DSM anxiety disorders in youth (32). Numerous studies and meta-analysis have examined the psychometric properties of the SCARED, indicating good psychometric properties for children and adolescents from various countries and on different language adaptations (32–34). The factorial structure of SCAARED shows a correspondence with the SCARED including four factors that correspond to the respective diagnostic categories of DSM-5, including agoraphobia, panic disorder, generalized anxiety, social anxiety, and separation anxiety disorder (29). The SCAARED has excellent internal consistency (α by Cronbach = 0.97).

In addition to its good psychometric properties, as eluded before, in contrast to the other available rating scales for anxiety disorders in adults which usually only include one anxiety disorder, the SCAARED includes all DSM-5 anxiety disorders. Moreover, the fact that the SCAARED was derived from the SCARED and share similar factors, allows to compare the scores of the two instruments between adults and youth and follow up studies from childhood into adulthood.

Many of the adult anxiety questionnaires noted above have been translated to Spanish [e.g.,: DASS-21 (35) STAI (36)], but not the SCAARED. Thus, the goals for this study were to: (1) To translate the (SCAARED) into Spanish and validate it in a non-clinical sample to verify its factor structure and its reliability (internal consistency and stability of the measurement); (2) To examine the concurrent validity of the Spanish translation of the SCAARED with the Depression, Anxiety and Stress Scale (DASS-21) (37) and the Beck Anxiety Inventory (BAI) (19) and (3) To analyze the construct validity by means of a factorial analysis to check the stability of the original model.

## Method

### Procedure and Participants

The sample comprised 131 college students (92.4% female, mean age 22 years old, all Caucasian), recruited from the University of Valencia, Spain, using non-probability and convenience sampling. Participants were informed about the PICCA project [Programa de Intervención Cognitivo-Conductual en Ansiedad (Cognitive Behavioral Intervention Program for Anxiety)], requesting their collaboration on a voluntary basis. Prior to data collection, the purpose, procedures, and expectations of the study were described to all participants All third-year students of the Faculty of Education (specialty of Therapeutic Pedagogy and Hearing and Language) and of the Faculty of Psychology and Speech Therapy (specialty of speech therapy), completed the Spanish version of the SCAARED at the same time they completed the BAI and the DASS-21. The administration of the battery was carried out during the rest time between two classes at the beginning of the second term of the 2019–20 academic year (first week of February). Participants completed the questionnaires independently, although in a collective/group session carried out in the presence of one of the investigators.

Following the completion of the questionnaires, we contacted the students who showed significant anxiety symptoms and agreed to be re-contacted by e-mail to participate in the clinical interview for confirmation of the diagnosis and, if appropriate, participate in PICCA. Participants who agreed returned for assessments at time 2 (15 days later) for administration retest reliability and diagnostic interview. The clinical interview was performed by a specialized psychologist (co-author of the study), administered a subset of relevant International Neuropsychiatric Interview chapters (38). This study was approved by the University of Valencia's Human Research Ethics Committee (H1549280336722). Consent was obtained from participants in accordance with the University of Valencia's Human Research Ethics Committee.

### Measures

The SCAARED (29) was translated, following the guidelines for translation and adaptation of Psychological Assessment instruments (39). After consulting the author and obtaining his consent, the English version of the questionnaire was initially translated by a bilingual psychologist, who proposed a first translation of the items into Spanish. In some cases, small adaptations were made since the literal translation could be misleading. A second translator performed the same task, to later reach a consensus on the modifications, and thus be able to propose a single translation. Finally, a back-translation into English was made, which was evaluated by the author of the scale to judge the adjustment of the terms used. The final version of the SCAARED in Spanish is the object of this study (see Supplementary Material 1).

To evaluate the validity (criteria, content, construct) of the SCAARED in Spanish, two existing anxiety self-reports were also administered. The DASS-21 is a short version derived from the full 42-item self-report scale DASS (37), which measures negative emotional states (Anxiety, Stress and Depression) with a selection of 7 elements from each construct. The DASS-21 has validated versions in Spanish, reporting adequate psychometric properties in the general adult population (40), in university students (22, 41), and in the clinical population (35). The Spanish version of the DASS-21 self-report instrument was used for this study (22).

The BAI (19) is a 21-item self-report instrument that is used for measuring the typical symptoms of anxiety disorders. It is designed to assess the severity of anxiety symptoms and is widely used in both clinical and research settings. Each item refers to symptoms experienced in the last week and is answered with a 4-point severity scale. The total score on the scale ranges from 0 to 63 points. There is a Spanish version (19) with excellent psychometric performance both in university students (42), in the general population general (43), and especially in the clinical population (44, 45).

### Data Analysis

The descriptive statistical analyses were performed with the SPSS (V26) licensed by the University of Valencia (Spain), the dimensionality analysis will be carried out with the software Mplus 8.3 (46). First, the descriptive statistical and the psychometric analysis of the SCAARED items was performed by calculating of mean, standard deviation, range, kurtosis, asymmetry, and corrected item-test correlation of all items on the scale and the internal consistency indicators (Cronbach's Alpha) and correlations with the other measures. The stability of the measurement (test-retest validity) was calculated with in a subsample of participants (*n* = 19) 15 days later. Receiver Operating Characteristic (ROC curve analysis) were used to determine SCAARED relative diagnostic accuracy. To analyze the structure of SCAARED a Confirmatory Factor Analysis (CFA) was developed on the original model (29). Additionally, we propose to perform an Exploratory Factorial Analysis (EFA) on the same data in order to venture a possible dimensionality different from the proposal in SCAARED original model.

## Results

The descriptive results of the 44 items of the SCAARED are shown in Table 1 in the Supplementary Material of this article (see Supplement S1). The value of the correlation between each item and the test shows low to medium values (0.11–0.60) and the indices of asymmetry and kurtosis that the distribution of response frequencies in the three item alternatives (Likert Type) show non-normal behavior. For the present validation study of the SCAARED in Spanish, we will consider the values of the original scale (29).

**Table 1:** Descriptive statistics of the 44 items of SCAARED.

	**Response categories**								
**Items**	**0**	**1**	**2**	**Mean**	**Standard Deviation**	**Range**	**Skewness**	**Kurtosis**	**Corrected item-test correlation**
1	47	59	26	0.84	0.732	2	0.260	−1.090	0.435
2	49	65	17	0.76	0.669	2	0.327	−0.780	0.474
3	65	48	18	0.64	0.713	2	0.651	−0.794	0.471
4	91	31	9	0.37	0.612	2	1.416	0.920	0.333
5	58	51	22	0.73	0.734	2	0.484	−1.009	0.320
6	103	24	4	0.24	0.498	2	1.935	3.003	0.331
7	20	81	30	1.08	0.615	2	−0.045	−0.337	0.462
8	12	47	77	1.50	0.661	2	−0.961	−0.211	0.452
9	63	46	22	0.69	0.745	2	0.581	−0.983	0.353
10	56	52	23	0.75	0.737	2	0.439	−1.048	0.561
11	73	40	19	0.58	0.723	2	0.832	−0.630	0.408
12	91	33	7	0.36	0.583	2	1.400	0.974	0.448
13	111	14	6	0.20	0.503	2	2.549	5.659	0.143
14	62	46	23	0.70	0.751	2	0.553	−1.031	0.237
15	114	14	3	0.15	0.420	2	2.847	7.845	0.472
16	74	41	16	0.56	0.703	2	0.871	−0.503	0.112
17	100	27	4	0.27	0.509	2	1.752	2.263	0.550
18	26	58	47	1.16	0.732	2	−0.260	−1.090	0.328
19	66	48	17	0.63	0.705	2	0.677	−0.738	0.415
20	71	41	19	0.60	0.730	2	0.778	−0.728	0.331
21	4	43	84	1.61	0.549	2	−1.019	0.024	0.472
22	57	51	23	0.74	0.740	2	0.457	−1.048	0.328
23	11	52	68	1.44	0.646	2	−0.712	−0.501	0.547
24	9	55	67	1.44	0.622	2	−0.655	−0.513	0.397
25	71	45	15	0.57	0.691	2	0.801	−0.546	0.466
26	99	24	8	0.31	0.580	2	1.765	2.073	0.223
27	72	41	18	0.59	0.722	2	0.809	−0.656	0.525
28	78	36	17	0.53	0.716	2	0.964	−0.415	0.413
29	24	60	47	1.18	0.718	2	−0.274	−1.018	0.454
30	34	68	29	0.96	0.695	2	0.051	−0.904	0.279
31	19	59	53	1.26	0.697	2	−0.403	−0.887	0.605
32	66	32	33	0.75	0.835	2	0.504	−1.384	0.379
33	17	56	58	1.31	0.692	2	−0.505	−0.817	0.274
34	43	51	37	0.95	0.783	2	0.081	−1.359	0.363
35	12	59	60	1.37	0.647	2	−0.527	−0.653	0.401
36	85	35	11	0.44	0.646	2	10.203	0.293	0.231
37	14	51	66	1.40	0.676	2	−0.681	−0.624	0.529
38	79	38	14	0.50	0.684	2	1.013	−0.210	0.486
39	29	64	38	1.07	0.715	2	−0.101	−1.019	0.539
40	89	27	15	0.44	0.692	2	1.299	0.304	0.382
41	39	50	42	1.02	0.789	2	−0.041	−1.387	0.512
42	70	44	17	0.60	0.710	2	0.769	−0.658	0.570
43	41	58	32	0.93	0.746	2	0.112	−1.184	0.325
44	37	61	33	0.97	0.733	2	0.048	−1.122	0.451

The Cronbach's alpha for the total SCAARED scale was adequate (α = 0.91), and very acceptable internal consistency for the items in each of the four dimensions of the scale (PA/SO α = 0.84; GA α= 0.85; SEP α= 0.62; SOC α= 0.91). As shown in Table 2, the test-retest correlations are high (<0.81) in all dimensions and in the total of the test. The *t*-tests for related samples show that the scores are stable 15 days after the first application.

**Table 2:** Means, standard deviations, Pearson's r_xy_ and t-student comparing test and re-test results for each of the SCAARED dimensions.

**SCAARED**	**First time**		**Second time**				
	**Mean**	**STD**	**Mean**	**STD**	**r_**xy**_**	***t***	***p***
Total	54.21	10.79	53.21	12.28	0.92	0.78	0.45
Panic disorder	16.28	7.31	16.21	6.50	0.95	0.11	0.91
Generalized anxiety disorder	22.36	2.95	21.50	3.82	0.87	1.71	0.11
Separation anxiety disorder	5.29	2.64	5.36	2.13	0.81	−0.17	0.86
Social anxiety disorder	10.29	3.29	10.00	3.39	0.86	0.34	0.74

Regarding concurrent validity, the DASS-21 and BAI tests were applied simultaneously to the SCAARED. Table 3 shows the correlation indices between the scores of the SCAARED dimensions and each of the tests used as criteria. Note that all correlations were significant (*p*-values ≤ 0.05) with the lowest being for Separation Anxiety.

**Table 3:** Correlations between DASS-21 and BAI tests and SCAARED dimensions (*N* = 131).

**SCAARED**	**DASS-21 stress**	**DASS-21 anxiety**	**DASS-21 depression**	**BAI**
TOTAL	0.67	0.73	0.60	0.73
Panic disorder	0.58	0.71	0.50	0.68
Generalized anxiety disorder	0.66	0.57	0.53	0.60
Separation anxiety disorder	0.26	0.34	0.19	0.42
Social anxiety disorder	0.30	0.36	0.39	0.36

*p-values ≤ 0.05*.

The diagnostic value was assessed by taking as a criterion having reached the cut-off point in the DASS-21 and in the BAI tests. The ROC curve was examined for each of the SCAARED subscales and for the total score. As shown in Table 4, the values of the area under the curve and the fitting model. The AUC Index value of the 0.88 total test can be considered very adequate. This is the same as the predicted values of AG (0.83) and TP/S (0.85). However, TAS (0.62) and AS (0.72) constructs are not properly adjusted. When analyzing the ROC curve data, the diagnostic contrast criteria used must be taken into account.

**Table 4:**
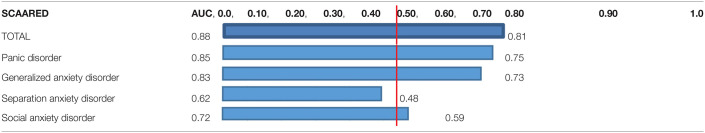
The Area Under the Curve (AUC) of the Receiver Operating Characteristic (ROC) curve and total fitting rates of the predictive model.

A confirmatory factorial analysis with Mplus version 8.3 (46) was conducted on the original model of SCAARED (29). The values of RMSEA (0.085), the Tucker Lewis Index (TLI = 0.621) and the comparative fit index (CFI = 0.641) are close to the critical values in each case, the SRMR (0.112) although the χ^2^ value is significant the final fit to the model (χ^2^ = 3,545.71; *p* = 0.00), point to an inadequate fit of the data to the original model.

Because the original structure was not confirmed, we carried out an EFA on the same sample to venture a possible dimensionality different from the proposal in the CFA. An EFA was performed on the 44 items and same sample. The Kaise-Meyer-Olkin index of sampling adequacy is 0.76 and Bartlett's sphericity test is significant (χ^2^ = 1,464.1; df = 861; *p* = 0.0), indicating that although the sample is small, we can proceed with the analysis (47).

The EFA was conducted to verify the four factor structure with Mplus. The values RMSEA (0.046), the TLI (0.92), CFI (0.93), SRMR (0.0866) and χ2 value (4,529.38; *p* = 0.00) indicate a good fit in the four-factor solution found and shown in Table 5. The first factor replicates the construct of Generalized Anxiety; the second factor rebuilds the construct of Panic Disorder. The third factor is defined by the items related to Social Anxiety and the fourth factor is defined by the items of Separation Anxiety.

**Table 5:** Factor Analysis for the four-factor solution (Saturations below 0.30 have been excluded).

***N* Item**	**Factor I**	**Factor II**	**Factor III**	**Factor IV**
	**Generalized anxiety disorder**	**Social phobia disorder**	**Panic disorder/significant somatic symptoms**	**Separation anxiety disorder**
21 I worry about things working out for me, [Le preocupa cómo le van a salir las cosas]	0.81			
08 It is hard for me to stop worrying, [Le cuesta dejar de preocuparse]	0.73			
23 I am a worrier, [Se preocupa demasiado]	0.68			
35 I worry about what is going to happen in the future, [Le preocupa de lo que vaya a pasar en el futuro]	0.66			
29 People tell me that I worry too much, [La gente le dice que se preocupa demasiado]	0.66			
37 I worry about how well I do things, [Se preocupa saber si está haciendo bien las cosas]	0.62	−0.31		
31 When I worry a lot, I feel restless, [Cuando se preocupa mucho, se siente inquieto(a)]	0.60			
07 I am nervous, [Estoy nervioso(a)]	0.53			
09 People tell me that I look nervous, [La gente me dice que parezco nervioso(a)]	0.50			
39 I worry about things that have already happened, [Me preocupo de las cosas que ya han sucedido]	0.46			
44 When I worry a lot, I feel irritable, [Cuando me preocupo mucho, me siento irritable]	0.46			
05 I worry about people liking me, [Me preocupa gustar a la gente]	0.35	−0.32		
24 When I worry a lot, I have trouble sleeping, [Cuando me preocupo mucho, tengo problemas para dormir]				
22 When I get anxious, I sweat a lot, [Cuando me siento ansioso(a), sudo mucho]				
34 I feel shy with people I don't know well, [Me siento tímido(a) con gente que no conozco bien]		−0.89		
27 It is hard for me to talk with people I don't know well, [Es difícil para mí hablar con gente que no conozco bien]		−0.85		
03 I don't like to be with people I don't know well, [No me gusta estar con personas que no conozco bien]		−0.83		
43 I am shy, [Soy tímido(a)]		−0.81		
10 I feel nervous with people I don't know well, [Me siento nervioso(a) con personas que no conozco bien]		−0.79		
42 I feel nervous when I go to parties, dances, or any place where there will be people that I don't know well, [Me siento nervioso(a) cuando voy a fiestas, bailes o cualquier lugar donde haya gente que no conozco bien]		−0.74		
41 I feel nervous when I am with other people and I have to do something while they watch me (for example: speak, play a sport), [Me siento nervioso(a) cuando estoy con otras personas y tengo que hacer algo mientras me miran (por ejemplo: hablar, hacer un deporte)]		−0.68		
17 I worry about going to work or school, or to public places, [Me preocupa ir al trabajo o a la universidad o instituto o a lugares públicos]		−0.46		
38 I am afraid to go outside or to crowded places by myself, [Tengo miedo de salir o ir a lugares concurridos solo(a)]		−0.44		0.38
14 I worry about being as good as other people, [Me preocupa ser tan bueno(a) como los demás]	0.34	−0.35		
01 When I feel nervous, It is hard for me to breathe, [Cuando me siento nervioso(a), me cuesta respirar]			0.70	
40 When I get anxious, I feel dizzy, [Cuando me pongo ansioso(a), me siento mareado(a)]			0.66	
06 When I get anxious, I feel like passing out, [Cuando me pongo ansioso(a), siento que voy a desmayarme]			0.66	
32 I am afraid of having anxiety (or panic) attacks, [Tengo miedo de tener ataques de ansiedad (o pánico)]			0.60	
19 I get shaky, [Me pongo tembloroso(a)]			0.56	
18 When I get anxious, my heart beats fast, [Cuando me siento ansioso(a), mi corazón late rápido]			0.55	
28 When I get anxious, I feel like I'm choking, [Cuando me siento ansioso(a), siento que me estoy ahogando]	0.36		0.52	
36 When I get anxious, I feel like throwing up, [Cuando me siento ansioso(a), tengo ganas de vomitar]			0.49	
12 When I get anxious, I feel like I'm going crazy, [Cuando me pongo ansioso(a), siento que me estoy volviendo loco(a)]			0.43	
15 When I get anxious, I feel like things are not real, [Cuando me pongo ansioso(a), siento que las cosas no son reales]			0.43	
02 I get headaches when I am at school, at work or in public places, [Tengo dolores de cabeza cuando estoy en la universidad, instituto, en el trabajo o en lugares públicos]			0.35	
25 I get really frightened for no reason at all, [Me asusto mucho sin ninguna razón]			0.31	
20 I have nightmares about something bad happening to me, [Tengo pesadillas sobre algo malo que me está pasando]				
26 I am afraid to be alone in the house, [Tengo miedo de estar solo(a) en la casa]				0.82
13 I worry about sleeping alone, [Me preocupa dormir solo(a)]				0.79
30 I don't like to be away from my family, [No me gusta estar lejos de mi familia]				0.50
04 I get nervous if I sleep away from home, [Me pongo nervioso(a) si duermo fuera de casa]				0.44
33 I worry that something bad might happen to my family, [Me preocupa que algo malo le pueda pasar a mi familia]				0.41
16 I have nightmares about something bad happening to my family, [Tengo pesadillas sobre algo malo que le pasa a mi familia]				0.40
11 I get stomachaches at school, at work, or in public places, [Me dan dolores del estómago en la universidad, instituto, en el trabajo o en lugares públicos]				0.32

*Factor I: Items 5, 7, 8, 9, 14, 21, 23, 28, 29, 31, 35, 37, 39, 44*.

*Factor II: Items 3, 5, 10, 14, 17, 27, 34, 37, 38, 41, 42*.

*Factor III: Items 1, 2, 6, 12, 15, 18, 19, 25, 28, 32, 36, 40*.

*Factor IV: Items 4,11, 13,16, 26, 30, 33*.

## Discussion

The aims of this study were to translate the SCAARED questionnaire (29) into Spanish and evaluate its psychometric properties in a sample of 131 college students. Overall, the results from the Spanish version of the SCAARED indicated good internal consistency (Cronbach α > 0.90), 2-week test-retest reliability (>0.86; *p* = 0.001), and adequate convergent validity with the DASS-21 and BAI. The results of the ROC analysis (AUC 0.88) inform us of excellent predictive value. The lowest correlation between the SCAARED and these instruments was with Separation Anxiety Disorder dimension because this disorder was only recently included in the DSM-5 as an adult anxiety disorder and as expected, except for the SCAARED, other anxiety self-reports do not include symptoms for this disorder.

The results of the Exploratory Factor Analysis showed four-factor structure (Generalized Anxiety, Social Anxiety, Panic Disorder/Significant Somatic Symptoms, and Separation Anxiety), which are consistent with the original SCAARED and correspond to the four factors reported for the instrument to screen for anxiety disorders in youth, the SCAARED (29). Moreover, the results of diagnostic validity, evaluated by means of the AUC indicators of the ROC curve were satisfactory. The above noted findings indicate that the Spanish version of the SCAARED behaves similarly to the English version and therefore appears to be an appropriate instrument for screening anxiety disorders in Spanish speaking adult populations. The fact that there are also Spanish versions of SCARED to screen youth for anxiety disorders (33, 34) with similar factorial structures, allows to use them as tools for evaluation of anxiety symptomatology in parents and their children and longitudinal studies of anxiety symptoms from childhood into adulthood.

Other adult anxiety measures available in Spanish are either dimensional (DASS-21; BAI; STAI, etc.) or specific to only one disorder (GAD-Q-IV, SPIN, LSAS, PDSS, etc.). In contrast, the SCAARED provides information on four types of anxiety disorders described in the DSM-5 and has excellent psychometric properties. In addition, it can be easily administered, is freely accessible, and time-effective (5–10 min). Finally, as noted above, the SCAARED can be crucial in obtaining and contrasting information from the patient/participant throughout life, since it bears similarities with the SCARED scale of which a Spanish version is already available (34).

Several limitations of our study are worth mention including a relatively small sample size, most of which were females and being a non-clinical sample which has impeded the calculation of some psychometric properties, such as inter-rater reliability and discriminatory validity. Consequently, further studies including larger samples and in clinical populations are needed. The present study consisted of the translation and adaptation of the scale and consequently, no qualitative studies (e.g., discussion groups) have been conducted on the comprehensibility of items in the Spanish. Nevertheless, the authors carefully reread the items in both languages to assess comprehensibility and changes were incorporated when consensus indicated that a change improved the translation. Likewise, during the administration of the questionnaire, special attention was paid to the evaluation of the meaning of each element, without giving rise to significant questions or observations on the part of the people participating in the study.

In summary, similar to the English version of the SCAARED, the Spanish version showed good psychometric properties suggesting that it is a potential tool to screen for DSM-5 anxiety disorders in non-clinical adult populations. Further studies in large samples of clinical populations are necessary to evaluate its sensitivity and specificity as well as cut-off points to screen for anxiety disorders.

## Data Availability Statement

The raw data supporting the conclusions of this article will be made available by the authors, without undue reservation.

## Ethics Statement

This study was approved by the University of Valencia's Human Research Ethics Committee (H1549280336722). The patients/participants provided their written informed consent to participate in this study.

## Author Contributions

All authors listed have made a substantial, direct and intellectual contribution to the work, and approved it for publication.

## Conflict of Interest

The authors declare that the research was conducted in the absence of any commercial or financial relationships that could be construed as a potential conflict of interest.

## References

[1] Kessler RC Angermeyer M Anthony JC de Graaf R Koen D Gasquet I. Lifetime prevalence and age-of-onset distributions of mental disorders in the World Health Organization's World Mental Health Survey Initiative. World Psychiatry. (2007) 6:168–76. 18188442 PMC2174588

[2] Polanczyk GV Salum GA Sagaya LS Caye A Rohde LA. Annual research review: a meta-analysis of the worldwide prevalence of mental disorders in children and adolescents. J Child Psychol Psychiatry. (2015) 56:345–65. 10.1111/jcpp.12381 25649325

[3] Remes O Brayne C van der Linde R Lafortune L. A systematic review of reviews on the prevalence of anxiety disorders in adult populations. Brain Behav. (2016) 6:e00497. 10.1002/brb3.497 27458547 PMC4951626

[4] Lijster JM Dierckx B Utens EM Verhulst FC Zieldorff C Dieleman GC. The age of onset of anxiety disorders: a meta-analysis. Can J Psychiatry. (2016) 62:237–46. 10.1177/0706743716640757 27310233 PMC5407545

[5] Essau CA Lewinsohn PM Lim JX Ho MR Rohde P. Incidence, recurrence and comorbidity of anxiety disorders in four majordevelopmental stages. J Affec Disord. (2018) 228:248–53. 10.1016/j.jad.2017.12.014 29304469 PMC5807150

[6] LeBlanc NJ Mackenzie B Aude H Anxiety disorders in emerging adulthood. In: Bui E Charney M Baker A editors. Clinical Handbook of Anxiety Disorders. Switzerland: Springer Nature Switzerland AG (2020). p. 157–73.

[7] Essau CA Lewinsohn PM Olaya B Seeley JR. Anxiety disorders in adolescents and psychosocial outcomes at age 30. J Affec Disord. (2014) 163:125–32. 10.1016/j.jad.2013.12.033 24456837 PMC4028371

[8] Rogers AH Wieman ST Baker AW Anxiety comorbidities: mood disorders, substance use disorders, and chronic medical illness. In: Bui E. Charney M Baker A editors. Clinical Handbook of Anxiety Disorders. Switzerland: Springer Nature Switzerland AG (2020). p. 77–104.

[9] Black JJ Rofey DL. An overview of common psychiatric problems among adolescent and young adult females: focus on mood and anxiety. Best Pract Res Clin Obstet and Gynaecol. (2018) 48:165–73. 10.1016/j.bpobgyn.2017.08.007 28970006

[10] Boden JM Fergusson DM Horwood LJ. Anxiety disorders and suicidal behaviours in adolescence and young adulthood: findings from a longitudinal study. Psychol Med. (2007) 37:431–40. 10.1017/S0033291706009147 17109776

[11] Ahmadpoor J Mohammadi Y Soltanian AR Poorolajal J Psychiatric disorders and associated risky behaviors among Iranian university students: results from the Iranian PDABs survey. J Public Health. (2020), 1–8. 10.1007/s10389-020-01229-8 30452701

[12] Dennis RE Boddington SJ Funnell NJ. Self-report measures of anxiety: are they suitable for older adults? Aging Mental Health. (2007) 11:668–77. 10.1080/13607860701529916 18074254

[13] Iglesias García C López García P Ayuso Mateos JL García JA Bobes J. Screening for anxiety depression in Primary Care: utility of 2 brief scales adapted to the new ICD-11-PC. Rev Psiquiatr Salud Ment. (2020) 30. S1888-9891(20)30014-8. 10.1016/j.rpsm.2019.12.001 34810133

[14] Balsamo M Cataldi F Fairfield B. Assessment of anxiety in older adults: a review of self-report measures. Clin Interv Aging. (2018) 13:573–93. 10.2147/CIA.S114100 29670342 PMC5896683

[15] Vasa RA Keefer A Reaven J South M White SW. Priorities for advancing research on youth with autism spectrum. J Autism Dev Disord. (2018) 48:925–34. 10.1007/s10803-017-3320-0 29164436

[16] Sandin B Chorot P Valiente RM. TCC de los Trastornos de Ansiedad: Innovaciones en niños y Adolescentes. Madrid: Klinic (2016).

[17] Simon E Bögels SM. Screening for anxiety disorders in children. Eur Child Adolesc Psychiatry. (2009) 18:625–34. 10.1007/s00787-009-0023-x 19415415 PMC2744785

[18] Spielberger C Gorsuch R Lushene R Vagg P Jacobs G. Manual for the State-Trait Anxiety Inventory. Palo Alto, CA: Consulting Psychologists Press (1983).

[19] Beck A Steer R Beck Anxiety Inventory Manual. San Antonio, TX: The Psychological Corporation (1993); Spanish version (2011).

[20] Muñiz J Fernández-Hermida JR La opinión de los psicólogos españoles sobre el uso de los tests. Papeles Psicól. (2010) 31:108–21. Available online at: http://www.papelesdelpsicologo.es/pdf/1801.pdf

[21] Sanz J Recomendaciones para la utilización de la adaptación española del Inventario de Ansiedad de Beck (BAI) en la práctica clínica. Clin Salud. (2014) 25:39–48. 10.1016/S1130-5274(14)70025-8

[22] Bados A Solanas A Andrés R Psychometric properties of the Spanish version of depression, anxiety and stress scales (DASS). Psicothema. (2005) 17:679–83. Available online at: http://www.psicothema.com/pdf/3165.pdf

[23] García-Campayo J Zamorano E Ruiz MA Pardo A Pérez-Páramo M López-Gómez V Freire. Cultural adaptation into Spanish of the generalized anxiety disorder-7 (GAD-7) scale as a screening tool. Health Qual Life Outcomes. (2010) 8:8. 10.1186/1477-7525-8-8 20089179 PMC2831043

[24] García-López LG Bermejo RM Hidalgo MD. The social phobia inventory: screening and cross-cultural validation in Spanish adolescents. Span J Psychol. (2010) 13:970–80. 10.1017/S1138741600002614 20977044

[25] Bobes J Badía X Luque A García M Paz González M Dal-Ré R.Validación de las versiones en español de los cuestionarios Liebowitz Social Anxiety Scale, Social Anxiety and Distress Scale y Sheehan Disability Inventory para la evaluación de la fobia social. Med Clin. (1999) 112:530–8. 10363239

[26] Santacana M Fullana MA Bonillo A Morales M Montoro M Rosado S. Psychometric properties of the Spanish sel-report version of the Panic Disorder Severity Scale. Compr Psychiatry. (2014) 55:1467–72. 10.1016/j.comppsych.2014.04.007 24850072

[27] Kendall P Compton S Walkup J Birmaher B Albano A Sherrill J.. Clinical characteristics of anxiety disordered youth. J Anxiety Disord. (2010) 24:360–65. 10.1016/j.janxdis.2010.01.009 20206470 PMC2838990

[28] American Psychiatric Association Diagnostic and Statistical Manual of Mental Disorders: DSM-5. Arlington, VA: Asociación Americana de Psiquiatría (2013).

[29] Angulo M Rooks BT Gill M Goldstein T Sakolsky D Goldstein. Psychometrics of the screen for adult anxiety related disorders (SCAARED)- A new scale for the assessment of DSM-5 anxiety disorders. Psychiatry Res. (2017) 253:84–90. 10.1016/j.psychres.2017.02.034 28359032 PMC5472098

[30] Birmaher B Khetarpal S Brent D Cully M Balach L Kaufman J. The Screen for Child Anxiety Related Emotional Disorders (SCARED): scale construction and psychometric characteristics. J Am Acad Child Adolesc Psychiatry. (1997) 36:545–53. 10.1097/00004583-199704000-00018 9100430

[31] Birmaher B Brent DA Chiappetta L Bridge J Monga S Baugher M. Psychometric properties of the Screen for Child Anxiety Related Emotional Disorders (SCARED): a replication study. J Am Acad Child Adolesc Psychiatry. (1999) 38:1230–36. 10.1097/00004583-199910000-00011 10517055

[32] Hale WW 3rd. Crocetti E. Raaijmakers QA Meeus WH. A meta-analysis of the cross-cultural psychometric properties of the Screen for Child Anxiety Related Emotional Disorders (SCARED). J Child Psychol Psychiatry. (2011) 52:80–90. 10.1111/j.1469-7610.2010.02285.x 20662993

[33] Canals J Hernández-Martínez C Cosi S Domènech E. Examination of a cutoff score for the Screen for Child Anxiety Related Emotional Disorders (SCARED) in a non-clinical Spanish population. J Anxiety Disord. (2012) 26:785–91. 10.1016/j.janxdis.2012.07.008 23023158

[34] Vigil-Colet A Canals J Cosi S Lorenzo-Seva U Ferrando PJ Hernández-Martínez C The factorial structure of the 41-item version of the Screen for Child Anxiety Related Emotional Disorders (SCARED) in a Spanish population of 8 to 12 years-old. Int J Clin Health Psychol. (2009) 9:313–27. Available online at: https://www.redalyc.org/pdf/337/33712028009.pdf

[35] Román F Santibáñez P Vinet EV Uso de las Escalas de Depresión Ansiedad Estrés (DASS-21) como Instrumento de Tamizaje en Jóvenes con Problemas Clínicos. Acta de Investig Psicol. (2016) 6:2325–36. 10.1016/S2007-4719(16)30053-9

[36] Buela-Casal G Guillén-Riquelme A Seisdedos Cubero N Cuestionario de Ansiedad Estado-Rasgo. Octava edición. Madrid: TEA Ediciones (2011).

[37] Lovibond P Lovibond S. The structure of negative emotional states: comparison of the Depression Anxiety Stress Scales (DASS) with the beck depression and anxiety inventories. Behav Res Ther. (1995) 33:335–43. 10.1016/0005-7967(94)00075-U 7726811

[38] Sheehan D Janavs J Baker R Harnett-Sheehan K Knapp E Sheehan M MINI International Neuropsychiatric Interview (5,0,0 version). Tampa, FL (2006). Available online at: http://www.iiap.es/files/mini.pdf (accessed January 12, 2020).

[39] Muñiz J Elosua P Hambleton R. Directrices para la traducción y adaptación de los tests: segunda edición. Psicothema. (2013) 25:151–57. 10.7334/psicothema2013.24 23628527

[40] Daza P Novy D Stanley M Averill P The depression anxiety stress scale-21: Spanish translation and validation with a Hispanic sample. J Psychopathol Behav Assess. (2002) 24:195–205. 10.1023/A:1016014818163

[41] Fonseca-Pedrero E Paino M Lemos-Giráldez S Muñiz J Psychometric properties of the Depression Anxiety and Stress Scales-21 (DASS-21) in Spanish college students. Ansiedad Estrés. (2010) 16:215–26.

[42] Sanz J Navarro ME Propiedades psicométricas de una versión española del Inventario de Ansiedad de Beck (BAI) en estudiantes universitarios. Ansiedad Estrés. (2003) 9:59–84. Available online at: https://www.researchgate.net/publication/285908290_Propiedades_psicometricas_de_una_version_espanola_del_Inventario_de_Ansiedad_de_Beck_BAI_en_estudiantes_universitarios

[43] Magán I Sanz J García-Vera MP. Psychometric properties of a Spanish version of the Beck Anxiety Inventory (BAI) in general population. Span J Psychol. (2008) 11:626–40. 10.1017/S1138741600004637 18988448

[44] Sanz J García-Vera M Fortún M El “Inventario de Ansiedad de Berck” (BAI): propiedades psicométricas de la versión española en pacientes con trastornos psicológicos. Psicol Conductual. (2012) 20:563–83. Available online at: https://www.behavioralpsycho.com/wp-content/uploads/2019/08/05.Sanz_20-3oa.pdf

[45] Vázquez-Morejón A Vázquez-Morejón R Bellido-Zanin BG. Beck anxiety inventory: psychometric characteristics in a sample from the clinical Spanish population. Span J Psychol. (2014) 17:E761 10.1017/sjp.2014.76 (accessed November 10, 2020). 26055653

[46] Muthén LK Muthén BO Mplus User's Guide. Los Angeles, CA: Muthén & Muthén (2017). Available online at: http://www.statmodel.com/download/usersguide/MplusUserGuideVer_8.pdf (accessed November 10, 2020).

[47] Cerny BA Kaiser HF. A study of a measure of sampling adequacy for factor-analytic correlation matrices. Multivariate Behav Res. (1977) 12:43–7. 10.1207/s15327906mbr1201_3 26804143

