# Supplementary material for: Psychometrics of the Spanish Version of the Screen for Adult Anxiety Related Disorders (SCAARED)
Source: Front Psychiatry. 2021 Feb 11;12:589422. doi: 10.3389/fpsyt.2021.589422 (PMC7904889; doi:10.3389/fpsyt.2021.589422)
Supplement: Supplementary file 1 [file Table_1.pdf]

SCREEN FOR ADULT ANXIETY RELATED EMOTIONAL DISORDERS (SCAARED) SPANISH VERSION

Versión para joven

Nombre: ____ Fecha: ______________________________

Edad: _____________________________ Genero: Masculino ☐ Femenino ☐

A continuación, hay una lista de oraciones que describen cómo se sienten las personas. Lee cada frase y decide cual es la respuesta que más se aproxima a lo que han sido sus sentimientos. ("Casi nunca es cierto o nunca es cierto", o" Es cierto algunas veces", o "Casi siempre cierto o siempre es cierto"). Luego, en cada oración, marque √ la casilla que corresponde a la respuesta que parece describirlo ahora o en los últimos 3 meses.

	**"Casi Nunca es Cierto" o "Nunca es Cierto"**	**"Es cierto Algunas Veces"**	**"Casi Siempre Cierto" o "Siempre es Cierto"**
1. Cuando me siento nervioso(a), me cuesta respirar.	0	1	2
2. Tengo dolores de cabeza cuando estoy en la universidad, instituto, en el trabajo o en lugares públicos.	0	1	2
3. No me gusta estar con personas que no conozco bien.	0	1	2
4. Me pongo nervioso(a) si duermo fuera de casa.	0	1	2
5. Me preocupa gustar a la gente	0	1	2
6. Cuando me pongo ansioso(a), siento que voy a desmayarme.	0	1	2
7. Estoy nervioso(a).	0	1	2
8. Me cuesta dejar de preocuparme.	0	1	2
9. La gente me dice que parezco nervioso(a).	0	1	2
10. Me siento nervioso(a) con personas que no conozco bien.	0	1	2
11. Me dan dolores del estómago en la universidad, instituto, en el trabajo o en lugares públicos.	0	1	2
12. Cuando me pongo ansioso(a), siento que me estoy volviendo loco(a).	0	1	2
13. Me preocupa dormir solo(a).	0	1	2
14. Me preocupa ser tan bueno(a) como los demás.	0	1	2
15. Cuando me pongo ansioso(a), siento que las cosas no son reales.	0	1	2
16. Tengo pesadillas sobre algo malo que le pasa a mi familia.	0	1	2
17. Me preocupa ir al trabajo o a la universidad o instituto o a lugares públicos.	0	1	2
18. Cuando me siento ansioso(a), mi corazón late rápido.	0	1	2
19. Me pongo tembloroso(a).	0	1	2
20. Tengo pesadillas sobre algo malo que me está pasando.	0	1	2
21. Me preocupa cómo me van a salir las cosas	0	1	2
22. Cuando me siento ansioso(a), sudo mucho	0	1	2

	"Casi Nunca es Cierto" o "Nunca es Cierto"	"Es cierto Algunas Veces"	"Casi Siempre Cierto" o "Siempre es Cierto"
23. Soy una persona preocupada.	0	1	2
24. Cuando me preocupo mucho, tengo problemas para dormir.	0	1	2
25. Me asusto mucho sin ninguna razón.	0	1	2
26. Tengo miedo de estar solo(a) en la casa.	0	1	2
27. Es difícil para mí hablar con gente que no conozco bien.	0	1	2
28. Cuando me siento ansioso(a), siento que me estoy ahogando.	0	1	2
29. La gente me dice que me preocupo demasiado.	0	1	2
30. No me gusta estar lejos de mi familia.	0	1	2
31. Cuando me preocupo mucho, me siento inquieto(a).	0	1	2
32. Tengo miedo de tener ataques de ansiedad (o pánico).	0	1	2
33. Me preocupa que algo malo le pueda pasar a mi familia.	0	1	2
34. Me siento tímido(a) con gente que no conozco bien.	0	1	2
35. Me preocupa lo que me vaya a pasar en el futuro.	0	1	2
36. Cuando me siento ansioso(a), tengo ganas de vomitar.	0	1	2
37. Me preocupa saber si hago las cosas bien.	0	1	2
38. Tengo miedo de salir o ir a lugares concurridos solo(a).	0	1	2
39. Me preocupo de las cosas que ya han sucedido.	0	1	2
40. Cuando me pongo ansioso(a), me siento mareado(a).	0	1	2
41. Me siento nervioso(a) cuando estoy con otras personas y tengo que hacer algo mientras me miran (por ejemplo: hablar, hacer un deporte).	0	1	2
42. Me siento nervioso(a) cuando voy a fiestas, bailes o cualquier lugar donde haya gente que no conozco bien.	0	1	2
43. Soy tímido(a).	0	1	2
44. Cuando me preocupo mucho, me siento irritable.	0	1	2

Angulo, M., Rooks, B. T., Gill, M., Goldstein, T., Sakolsky, D., Goldstein, B., . . . Birmaher, B. (2017). Psychometrics of the screen for adult anxiety related disorders (SCAARED)- A new scale for the assessment of DSM-5 anxiety disorders. Psychiatry Research, 253,84-90. http://doi.org/10.1016/j.psychres.2017.02.034

Translation and adaptation into Spanish:

Sánchez-Cueva S, Alonso-Esteban Y, Sánchez-Cueva P, Birmaher B and Alcantud-Marín F (2021) Psychometrics of the Spanish Version of the Screen for Adult Anxiety Related Disorders. Front. Psychiatry 12: 589422 doi: https://doi.org/10.3389/fpsyt.2021.589422
